# Health risk factors, status and service utilisation of adults in primary health care settings in Qatar: The HEALTHSIGHT study protocol

**DOI:** 10.1371/journal.pone.0304160

**Published:** 2024-05-29

**Authors:** Mohamed Ahmed Syed, Mariam Hassan, Shajitha Thekke Veettil, Tamara Marji, Hanan Khudadad, Dana Bilal El Kaissi, Abduljaleel Abdullatif Zainel, Hafiz Ahmed Mohamed, Bindya Mathew, Muslim Abbas Syed, Ahmed Sameer Alnuaimi

**Affiliations:** Department of Clinical Research, Primary Health Care Corporation, Doha, Qatar; University of Rome Tor Vergata: Universita degli Studi di Roma Tor Vergata, ITALY

## Abstract

**Background:**

The emergence of non-communicable diseases (NCDs) has been well documented in recent literature which constitute a significant global burden of disease. Qatar which has a significantly high prevalence of NCDs with early on set. Epidemiological and health service utilization information plays a central role in facilitating informed decision making and application of the fundamental principles of PHC in planning and delivery of healthcare with aim to prevent and control NCDs. To enable this, the Department of Clinical Research at Primary Health Care Corporation (PHCC), Qatar’s publicly funded and largest primary care provider designed the Health Assessment Linking Trends in Health Status, Risks, and Healthcare Utilization (HEALTHSIGHT) study. This paper describes the HEALTHSIGHT study protocol.

**Methods:**

The proposed study will use a cross sectional study design involving a random sample of participants enrolled across all 31 PHCC health centers. Individuals aged 18 and above years old registered with PHCC and hold a valid health card and contact information on PHCC’s electronic medical records (EMR) will be eligible for inclusion. A stratified random sample not proportional to size sampling technique will be employed to obtain a representative sample size of the PHCC population (N = 6000). Participants will be scheduled for an appointment at a PHCC health centre where a data collector will obtain informed consent, collect vital sign information and administer a questionnaire. A phlebotomist will collect a blood sample. Health service utilization data will be extracted from PHCC’s EMR.

**Discussion:**

Epidemiological and health service utilization information is essential to plan and monitor primary care and public health services. The HEALTHSIGHT study, with the help of a randomly selected representative sample from Qatar’s primary healthcare settings, provides a unique opportunity to capture this information. This study design will closely represent a real-world understanding of the health risk, status and utilisation and is likely to provide important data to guide primary care planning and delivery in Qatar. The proposed protocol provides an example of a robust nationwide study that be undertaken in short duration using limited resource which can be undertaken in other similar settings.

## Background

The emergence of non-communicable diseases (NCDs) has been well documented in recent literature which constitute a significant global burden of disease [1, 2]. According to Word Health Organization (WHO) it is estimated that NCDs cause 40 million deaths annually out of which 80% are due to cardiovascular disease, chronic obstructive pulmonary disease, diabetes mellitus and cancers [2, 3]. The prevalence of NCDs such as cardiovascular disease and diabetes mellitus is particularly high in the Gulf Cooperation Council countries. This includes Qatar which has a significantly high prevalence of NCDs with early on set [4]. The country has one of the highest prevalence of diabetes worldwide and a high proportion of people visiting NCD clinics in primary care settings have diabetes [5, 6].

It is important to acknowledge that there are modifiable and non-modifiable risk factors associated with NCDs which can serve as important indicators for disease surveillance, mapping and projecting future trends and devising treatment and preventive strategies [7, 8]. The commonly known non-modifiable risk factors include genetic predisposition, family history age, gender, and socio-economic status [9]. Whereas the modifiable behavioral risk factors which are also known as lifestyle risk factors mainly encompass diet, physical inactivity, obesity, tobacco use, excessive alcohol consumption, psychosocial factors [10, 11]. Moreover, there are modifiable physiological risk factors (which are also termed as metabolic risk factors) which entail hypertension, dyslipidemia, hyperglycemia, and chronic kidney disease [12–14]. Furthermore, evidence suggests that the wider determinants of health such as socio-economic, cultural, political, and environmental determinants, including population aging, globalization, urbanization, and the accompanied nutrition transition has also significantly contributed towards the recent rise in NCDs globally [9].

The WHO’s proposed a model of primary health care (PHC) after the ‘health for all’ initiative [15]. The concept is based on a multi-sectoral approach and aims to deliver comprehensive care which is well suited in addressing NCDs and its prevention [16–18]. Primary health care over the last few decades has established itself as the cornerstone for building a strong healthcare system that ensures better health outcomes, lower costs, and enables greater equity in health. In its true essence it must cater comprehensive and people-centered healthcare tailored to meet the specific needs of the communities and populations [15]. This should consider individuals of all age groups, ethnicities, and cultural backgrounds. Moreover, the model should ideally incorporate and coordinate health promotion, apply principles of preventive medicine, promote acute and chronic care management activities with the prime objective to deliver equitable access and safe high-quality care. Hence, strengthening primary health care to improve health outcomes and reduce the prevalence of NCDs is essential [14–16]. Epidemiological and health service utilization information plays a central role in facilitating informed decision making and application of the fundamental principles of PHC in planning and delivery of healthcare with aim to prevent and control NCDs. To enable this, the Department of Clinical Research at Primary Health Care Corporation (PHCC), Qatar’s publicly funded and largest primary care provider designed the Health Assessment Linking Trends in Health Status, Risks, and Healthcare Utilization (HEALTHSIGHT) study. This paper describes the HEALTHSIGHT study protocol.

## Methods

### Aim

The HEALTHSIGHT study will provide epidemiological information on the health risk factors, status and health service utilisation in adult population registered at PHCC, with an aim to facilitate planning and monitoring of primary care health services in Qatar.

### Study setting

Qatar is a peninsular Arab country. The country in the recent years has invested significantly towards universal publicly funded health care system for Qataris and non-Qatari residents. PHCC is Qatar’s largest primary care provider. It delivers comprehensive, integrated and coordinated person-centered health care services in the community by focusing on disease prevention, healthy lifestyles and wellness. As of June 2023, approximately 1.7 million individuals were registered at PHCC across its 31 health centers.

### Study design and population

The proposed study will use a cross sectional study design involving a random sample of participants enrolled across all health centers of PHCC. Individuals aged 18 and above years old registered with PHCC and hold a valid health card and contact information on PHCC’s electronic medical records (EMR) will be eligible for inclusion. Individuals with difficulties related to mobility and communication, bleeding disorders and mental disabilities or pregnant will be excluded.

### Sample selection

A stratified random sample not proportional to size sampling technique will be employed to obtain a representative sample size of the PHCC population. A full list of adult individuals aged 18+ years registered at PHCC will be extracted and stratified into 60 strata. Strata will be defined based on five age groups (18–29, 30–39, 40–49, 50–59 and 60 + years), gender (male and female) and the six major nationality categories (Qatari, North African, South East Asian, Southern Asian, Western Asian and others) based on geographical regions.

A valid relative frequency estimate was calculated for each of the 60 strata individually, while maintaining the representation of the population estimates using a weighting approach based on the sampling fraction. Error margins were calculated for 95% confidence intervals for five values (sample proportions of 10%, 20%, 30%, 40%, 50%). In addition, it was calculated for various types of samples to establish the smallest and largest error margin. Both calculations showed error margins ranging from 0.8% to 9.8%. Based on these, a fixed strata size of 100 is agreed to obtain a total sample size of 6000. Similar studies conducted at PHCC have has a response rate of approximately 10%. Hence the target sample size was increased to 60,000 to account for the non-response rate.

### Participant recruitment

**Fig 1:**
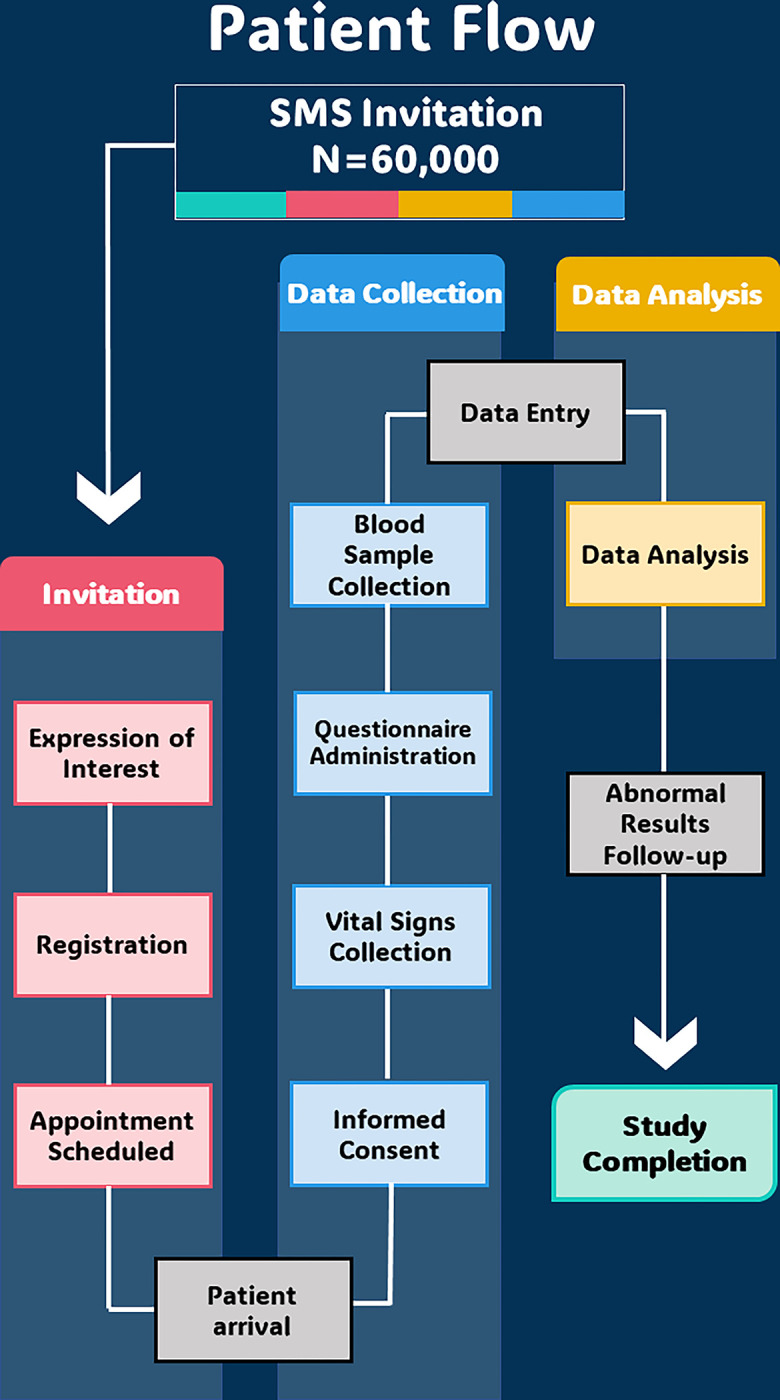
HEALTHSIGHT study patient flow.

The target sample of 60,000 individuals will randomly be extracted from PHCC’s electronic medical records system with their health record number, name, age, gender, nationality and mobile phone number–See Fig 1. Participants will be sent Short Message Service (SMS) messages on their mobile phones inviting them to express their interest to participate in the study via an online form. Individuals expressing interest will be contacted to schedule an appointment at a health centre.

### Data collection

Trainer data collectors will be allocated at each PHCC health centre. At their scheduled appointment, the data collector (PHCC nurse) will escort the participant to a private room at the health centre to register them on PHCC’s EMR system, obtain informed consent, collect vital sign information and administer a questionnaire. Following this, the data collector will escort the participant to the phlebotomy room where a trained PHCC phlebotomist will collect a blood sample.

The study will collect the below listed data:

- Vital sign measurements: Height, weight, hip and waist circumference and blood pressure measurements.
- Questionnaire: Sociodemographic (education level, marital status, number of children, housing status, employment status and household income) behavioural risk factors (physical activity, smoking status, fruit and vegetable consumption and oral hygiene) and health status information (existing diagnosis and treatment of NCDs, family history of angina or myocardial infarction, perceived health status and disabilities)
- Blood sample: Aspartate transaminase and alanine transaminase to measure liver function, creatinine to measure kidney function, complete blood count, ferritin and total iron-binding capacity test receptors to diagnose anaemia, glycated haemoglobin to indicate poor control or undiagnosed diabetes, Serum albumin to assess liver disease and kidney disorders, Total serum cholesterol, low density lipoprotein-cholesterol, high density lipoprotein-cholesterol to diagnose dyslipidemia
- EMR: health service utilisation data for 12 months will be extracted from PHCC’s EMR. This will include:
  ○ Demographic characteristics: Age, gender, nationality, registered health centre and zone of residence.
  ○ Diagnosis of NCD risk factor end points (Hypertensive heart disease, Hemorrhagic Stroke, Coronary Heart Disease, Atherothrombotic Stroke, Peripheral Vascular Disease, Type 2 Diabetes and Chronic Obstructive Pulmonary Disease)
  ○ Physician consultation details (number of consultations and for reason for consultation)
  ○ Dentist consultation details (number of consultations and for reason for consultation)
  ○ Laboratory investigation details (number of investigations and type of investigations)
  ○ Prescriptions details (number of prescriptions and type of medication)
  ○ Radiology details (number of investigations and type of investigations)
  ○ Physiotheraphy details (number of sessions and reasons for session)

### Analytical plan

Prevalence rates with 95% confidence interval (CI) for socioeconomic, behavioral, and metabolic health risk factors and report them stratified by age, gender, nationality, health centre and area of residence with 95% CI. In addition, prevalence of NCDs and health service utilization data will be described stratified by age, gender, nationality, health centre and area of residence using frequencies and percentages. Chi-square test of independence will be used to assess the statistical significance of associations between risk factors, health status and resource utilization with sociodemographic factors.

**Fig 2:**
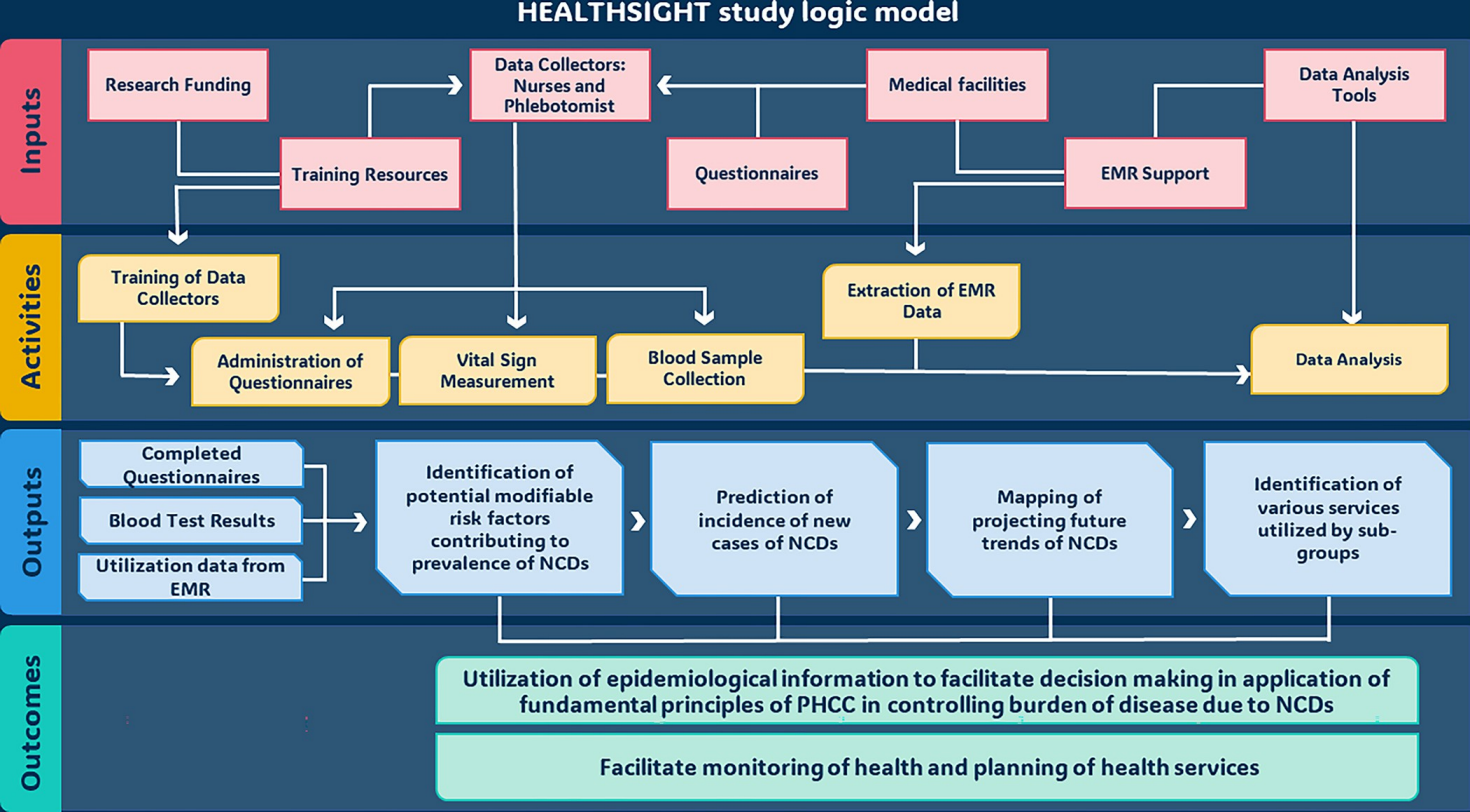
HEALTHSIGHT logic model.

A logic model of the protocol is presented in Fig 2.

### Ethics approval

The study was reviewed and approved by PHCC’s Independent Review Board (BUHOOTH-D-23-00058). Informed assent will be obtained from participants aged 18 or over. Overall, the study will be planned and conducted with integrity according to generally accepted ethical principles.

## Discussion

Epidemiological and health service utilization information is essential to plan and monitor primary care and public health services. The HEALTHSIGHT study, with the help of a randomly selected representative sample from Qatar’s primary healthcare settings, provides a unique opportunity to capture this information. This study design will closely represent a real-world understanding of the health risk, status and utilisation and is likely to provide important data to guide primary care planning and delivery in Qatar.

The findings of the study will help identify the potential risk factors (modifiable) contributing to the NCDs and predict incidence of new cases which will be substantiated by recording the metabolic risk factors. These indicators can also aid in mapping and projecting future trends of the NCDs. Moreover, Qatar has a diverse expatriate population with local Qatari’s constituting of approximately 12% of the total population. The study will help identify the frequency of the health services utilized by the patients accessing the various health centers. This information is vital in prioritization of the health services which are more inclined towards the needs of the patients promoting a patient centered approach.

WHO is a strong advocate of surveillance of ongoing NCDs and the associated risk factors. This protocol is in accordance with the recommendations of the WHO and moreover aims to project future trajectories and disease patterns which will aid in devising mitigation and preventive strategies within a well-established primary health care system of Qatar. Similarly, a study was conducted in 7 provinces of Nepal to assess the distribution and determinants of NCDs utilizing a WHO recommended STEPS survey(8). The surveillance data reported a significant proportion of Nepalese population exposed to different risk factors associated with NCDs. Our study is prospective longitudinal study, and we aim to repeat the study every three years which will significantly contribute towards the monitoring of specific NCD prevention and control interventions and planning of health services throughout the primary health centers (constituting the Primary Healthcare Cooperation) in Qatar.

Some of the limitations of the study include that the participants will be invited to participate in the study by short messaging service through mobile phones. Similar studies have reported to collected epidemiological data pertaining to NCDs by conducting national surveys. It may be a potential barrier as there is a risk that patients with low literacy levels may not fully comprehend the information provided through the text message and there may be low response rate. However, the study ensures that the participants make informed decision of participating in the study and the recruitment process will also be facilitated by the healthcare staff working in the different healthcare centers. The key strength of the study is that it’s the first of its kind in the country that aims to capture rich epidemiological data which will help identify the potential risk factors (modifiable) contributing to the prevalence of NCDs and predict incidence of new cases and map and project future trends of the NCDs in the country among the different sub-groups of population. Majority of similar studies to date are based on a non-random sample, focus on specific population groups making it difficult to generalise findings or resource intensive. Generally, healthcare systems invest significant resources in conducting NCD surveillance studies. The proposed protocol provides an example of a robust nationwide study that can be undertaken in a short duration using limited resource. This will be useful for healthcare systems seeking to e undertaken such studies in similar settings and can be repeated more frequently to provide recent information to support public health decision making.

## List of abbreviations

EMR: Electronic Medical Records
HEALTHSIGHT: Health Assessment Linking Trends in Health Status, Risks, and Healthcare Utilization
NCD: Non-Communicable Diseases
PHC: Primary Health Care
PHCC: Primary Health Care Corporation
WHO: Word Health Organization
